# Effect of Increasing Levels of Web-Based Behavioral Support on Changes in Physical Activity, Diet, and Symptoms in Men With Prostate Cancer: Protocol for a Randomized Controlled Trial

**DOI:** 10.2196/11257

**Published:** 2018-11-15

**Authors:** Kerri M Winters-Stone, Stacey A Kenfield, Erin L Van Blarigan, Esther L Moe, Justin W Ramsdill, Kimi Daniel, Greta Macaire, Kellie Paich, Elizabeth R Kessler, Omer Kucuk, Theresa W Gillespie, Karen S Lyons, Tomasz M Beer, Jeanette M Broering, Peter R Carroll, June M Chan

**Affiliations:** 1 Knight Cancer Institute Oregon Health & Science University Portland, OR United States; 2 School of Nursing Oregon Health & Science University Portland, OR United States; 3 Department of Urology University of California, San Francisco San Francisco, CA United States; 4 Department of Epidemiology & Biostatistics University of California, San Francisco San Francisco, CA United States; 5 Department of Medicine Oregon Health & Science University Portland, OR United States; 6 Oregon Clinical Translational Research Institute Oregon Health & Science University Portland, OR United States; 7 Helen Diller Comprehensive Cancer Center University of California, San Francisco San Francisco, CA United States; 8 Movember Foundation Culver City, CA United States; 9 University of Colorado School of Medicine University of Colorado Cancer Center Aurora, CO United States; 10 Winship Cancer Institute of Emory University Atlanta, GA United States; 11 William F Connell School of Nursing Boston College Boston, MA United States

**Keywords:** prostatic neoplasm, survivorship, diet, exercise, internet, behavior, text messaging, accelerometry

## Abstract

**Background:**

More than 3.1 million men in the United States are prostate cancer survivors. These men may improve their physical function, quality of life, and potentially their prognosis by adopting healthier lifestyle habits. The internet provides a scalable mechanism to deliver advice and support about improving physical activity and dietary habits, but the feasibility and acceptability of a Web-based lifestyle intervention and the dose of support necessary to improve health behaviors are not yet known.

**Objectives:**

The Community of Wellness is a Web-based intervention focused on supporting exercise and healthy dietary practices for men with prostate cancer. The objectives of this study were to determine the feasibility, acceptability, and preliminary efficacy of the Community of Wellness Web portal among prostate cancer survivors by conducting a randomized controlled trial (RCT) comparing 4 levels of additive Web-based content and interaction with participants: Level 1 (Teaching; Control), Level 2 (Teaching + Tailoring), Level 3 (Teaching + Tailoring + Technology), and Level 4 (Teaching + Tailoring + Technology + Touch).

**Methods:**

This is a single-blinded RCT comparing 3 levels of behavioral support within the Community of Wellness Web portal intervention (Levels 2 to 4) with each other and with the control condition (Level 1). The control condition receives general static Web-based educational information only on physical activity and dietary habits, self-efficacy for behavior change, motivation for physical activity, and changes in anxiety and treatment-related side effects. We will enroll and randomize 200 men with prostate cancer equally to 4 levels of the Community of Wellness Web-based intervention for 3 months (50 men per level). Surveys will be completed by self-report at baseline, 3 months (immediately postintervention), and 6 months (3 months postintervention). Feasibility and acceptability will be assessed by enrollment statistics, Web-based usage metrics, and surveys at the 3-month time point. We will also conduct focus groups after the postintervention follow-up assessment in a sample of enrolled participants to evaluate elements of usability and acceptability that cannot be obtained via surveys.

**Results:**

Enrollment is ongoing, with 124 enrolled. Study completion (6-month follow-up) is expected by July 2019.

**Conclusions:**

The goal of the study is to identify the level of support that is feasible, acceptable, promotes behavior change, and improves health in men with prostate cancer to inform future efforts to scale the program for broader reach.

**Trial Registration:**

ClinicalTrials.gov NCT03406013; https://clinicaltrials.gov/ct2/show/NCT03406013 (Archived by WebCite at http://www.webcitation.org/73YpDIoTX).

**International Registered Report Identifier (IRRID):**

PRR1-10.2196/11257

## Introduction

Nearly 3 million men in the United States are living with prostate cancer [1], and median survival time following diagnosis for these older men is 16 years [1-3]. During this time, men with prostate cancer might experience adverse effects of aging and persistent side effects of cancer treatment that reduce their physical function, quality of life (QoL), and potentially their prognosis [4-8]. A cancer diagnosis is a *teachable moment* when individuals are often motivated to change behavior to reduce risk of adverse health outcomes and optimize QoL [9-16]. However, advice provided is often inconsistent, and the current standard of care does not support resources for lifestyle behavior change for men with prostate cancer in the United States [17].

For men with prostate cancer, regular physical activity is an excellent strategy to offset age- and treatment-related declines in physical functioning, mental health, and QoL [18]. We and others have shown that when prescribed appropriate physical activity in the form of either aerobic and/or resistance exercise training, it might manage acute and chronic treatment-related symptoms and side effects [10,19-22]. Furthermore, recent observational evidence from our team and others suggests that engaging in sufficient amounts of aerobic activities may reduce prostate cancer progression [11] and prostate cancer–specific death [23-27]. Despite the evidence for physical activity benefits in men with prostate cancer, more than 75% of prostate cancer survivors fail to achieve recommended amounts of aerobic exercise and only 4% engage in any resistance exercise [28]. Prostate cancer survivors may fail to meet the physical activity recommendations because they are not receiving information about the effects of specific types of exercise on the health outcomes that are most important for them. A recent study reported that prostate cancer survivors on androgen deprivation therapy regard a physician’s general recommendation to be physically active as important; however, they are frustrated when providers lack specific knowledge about the type of exercise they should do and lack knowledge on how exercise specifically affects their cancer and related health issues [29-33].

In addition, accumulating evidence from our team suggests that several dietary factors may reduce the risk of prostate cancer progression and death, including greater intakes of cruciferous vegetables, vegetable fat, fish, and cooked tomatoes and lower intake of whole milk and poultry with skin [11,34-43]. Many of these dietary factors (eg, cruciferous vegetables, cooked tomatoes, and whole milk) appear to have specific associations with prostate cancer progression and are not included in general nutrition guidelines for cancer survivors [10]. Furthermore, excess supplementation of vitamins or minerals may increase risk of prostate cancer progression, yet cancer survivors, including men with prostate cancer, report high use of dietary supplements [44,45].

Successful interventions that promote healthy lifestyles can be challenging to scale to the broader population and may not readily reach those persons who are most in need [46]. Center-based lifestyle programs specific to cancer survivors are rare and, if available, tend to be offered only in major academic medical centers in urban areas, whereas home-based interventions have been tested in other cancers [47] including prostate cancer [48]. They are rarely comprehensive enough to address both physical activity and dietary change and raise challenges for tailoring programs to individual needs, maintaining safety and efficacy, and sustaining motivation, especially when counseling is an integral part and would be difficult to maintain indefinitely [49,50]. For optimal engagement, there is a need to provide appropriate content, accommodations, and reinforcement in a way that can successfully promote behavior change [51]. The internet provides a potentially scalable and economical way of delivering lifestyle interventions to cancer survivors [52]. There is 1 Web-based trial (Prostate 8) focused on diet, exercise, and not smoking that has been fully enrolled and will be published soon (NCT02470936). No studies published to date are specific to men with prostate cancer and none simultaneously address physical activity and dietary change while elucidating the types and levels of behavioral support that are effective [53]. For example, men with prostate cancer cite lack of specific guidance for exercise and lack of motivation as barriers to physical activity [33]; thus, interventions that attempt to systematically remove these barriers might be particularly effective but have not yet been evaluated. Another important consideration for future implementation and scalability is identifying the level of *intensity* of an intervention that produces a meaningful benefit. In their translational model of survivorship care, for example, Alfano et al call for trials that evaluate whether or not developing and delivering a survivorship care plan alone for survivors is enough to improve their outcomes or if a more interactive process is necessary [54].

On the basis of the evidence indicating that diet and physical activity habits might offer benefits for men living with prostate cancer, we developed a multisite, national, pilot feasibility study to build and test a Web-based interactive lifestyle management program, the “Community of Wellness,” to provide tools and support for men with prostate cancer to optimize their health through physical activity and diet. It is unclear what types and how much support men may need to make meaningful changes using a Web-based tool; therefore, the objective of this project is to identify optimal combinations of different types and levels of support for diet and physical activity behavior change. To address this objective, we will conduct a randomized controlled trial (RCT) to establish the feasibility and acceptability of the Web-based intervention and explore the preliminary efficacy of levels of support to improve diet and physical activity behaviors, motivational behaviors, and symptoms and side effects associated with prostate cancer and its treatment.

## Methods

### Study Design and Setting

The “Community of Wellness” study is supported by the Movember Foundation and is part of their broader initiative—*TrueNTH USA.* The study is a single blinded, parallel 4-arm RCT comparing 3 levels of increasing behavioral support with a usual care group that receives Web-based general educational information only. Although participants will know which study arm they are randomized to, outcomes are based on self-reported surveys completed by men online; thus, analysts can remain blinded to group assignment. We aim to enroll and randomize 200 men with prostate cancer equally to 1 of the 4 study arms for 3 months. Men complete surveys online at baseline, 3 months (postintervention), and 6 months (3 months postintervention). We will also conduct focus groups after the postintervention follow-up assessment in a subsample of enrolled participants to evaluate elements of usability and acceptability that cannot be obtained via standardized surveys.

### Study Population

The study population is men with a history of prostate cancer. Men are eligible to participate if they self-report receiving a prostate cancer diagnosis, are aged 18 years or older, able to read English on a computer screen, and have a personal device that has internet access and text messaging and a personal email address. Men are not eligible to participate if they report any contraindications to exercise based on the American College of Sports Medicine exercise preparticipation screening criteria [47] and do not receive a physician clearance to participate in moderate intensity physical activity. In addition, men who are currently receiving chemotherapy or radiation therapy will be required to receive a physician clearance before enrollment. Eligibility is assessed via phone screening, and eligible men provide consent online.

Consented participants are randomly assigned to 1 of the 4 Levels of intervention (n=50 per group). Recruitment is based out of 4 academic medical centers (Oregon Health & Science University [OHSU, primary study coordinating center], University of California San Francisco [UCSF], University of Colorado Denver [UC Denver], and Emory University). The primary recruitment approach will use patient databases to identify potential participants who will be mailed a letter and study brochure that directs them to contact research staff at the coordinating center to learn more about the study. In addition to this approach, clinics will have study brochures in waiting and exam rooms. Once deemed eligible, men are emailed a link to provide consent and complete surveys online, and once these are completed, they receive a separate link to access the Web portal where they will access their randomized level of the intervention. The study is approved by 3 institutional review boards at OHSU, UCSF, and UC Denver and is under review at Emory University.

### Study Arms

Our overarching goal is to support healthy diet and physical activity among men with prostate cancer; thus, we aim for a program that has strong acceptability and feasibility and which is also effective and can be broadly disseminated. The intervention was informed by the social cognitive theory that focuses on individual behavior change by including intervention components that addressed key constructs of self-control, expectations, and behavioral capability by including information about exercise for cancer survivors and tailored recommendations for diet and exercise, observational learning by including exercise videos and diet recipes, reinforcement by including technological components that provided feedback on goals, and promotion of self-efficacy through the use of health coaches [55]. We also incorporated techniques to promote user engagement with digital interventions by including credible information that was tailored to address the health needs of prostate cancer survivors and their desire to have information about health behaviors that are specific to their disease [33] as well as the use of prompts to facilitate motivation [56]. The intervention is structured around supporting 3 specific physical activity recommendations (aerobic, strength, and flexibility/balance exercise) that align with recommendations from the American College of Sports Medicine and 8 dietary recommendations (intake of cruciferous vegetables, cooked tomatoes, fish, processed meat, poultry with skin, whole milk, vegetable fat, and vitamin supplements) that are consistent with the scientific literature on diet and prostate cancer at the time of the study’s development (approximately 2016).

**Table 1:** Intervention components available from the Community of Wellness Web portal by level.

Level^a^	Type of support	Component^b^
1	Teaching; educational information about exercise and diet, presented in a static website format	Exercise guidelines for prostate cancer survivors^b^; diet guidelines for prostate cancer survivors^b^; exercising safely with various health issues^b^; and resources (exercises and recipes for recommended foods)^b^
2	Tailoring; Level 1 plus personalized exercise and diet advice	Personalized exercise prescription and diet recommendation^b^ and videos of recommended exercises^b^
3	Technology; Levels 1 to 2 plus technology support	Text messaging to support and reinforce diet and physical activity behaviors; Fitbit Alta plus physical activity progress reports based on physical activity tracker data^b^; and self-report log of progress toward diet and exercise goals^b^
4	Touch; Levels 1 to 3 plus *live* personal support from a health coach	Phone consult with diet and exercise coaches (30 min each) and ongoing Web-based (via email) support from diet and exercise coaches

^a^Each progressive level receives the intervention components of the level before it; for example, a participant randomized to Level 3 technology receives all the components of Levels 1 to 3 but not 4.

^b^Items available on the Web portal.

Each study arm provides additive levels of behavioral support accessed via a Web portal that can be viewed on multiple computing devices including a personal computer, tablet, or smartphone. The 4 study arms are as follows: Teaching (Level 1; usual care control), Tailoring (Level 2), Technology (Level 3), and Touch (Level 4). Each level receives the tools and resources of the prior level such that Level 4 participants receive the most intervention components and is the only level to include additional personal contact via phone and email between a participant and a health coach. Table 1 summarizes the intervention components that are accessible to men who are randomized to each of the 4 arms.

The intervention components are described in more detail below.

#### Level 1: Teaching

This level contains Web-based static information about physical activity and diet advice for men with prostate cancer.

#### Level 2: Teaching + Tailoring

**Figure 1:**
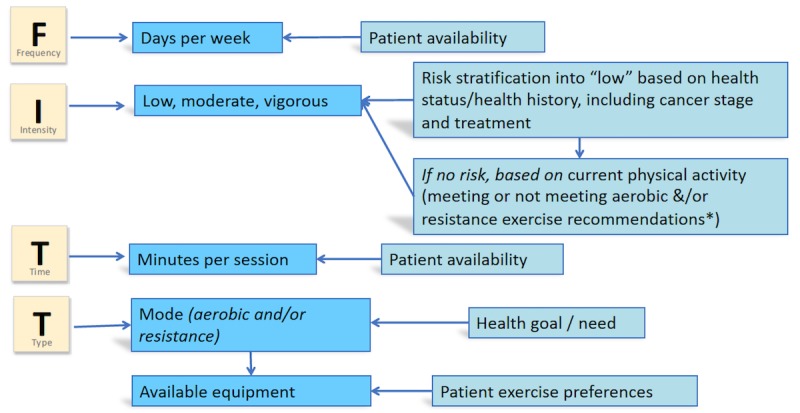
Algorithm to generate a tailored exercise prescription based on participant input. *Current physical activity determined by answers to the Rapid Assessment of Physical Activity (PA) questionnaire that determines how regularly men participate in moderate or vigorous aerobic exercise and resistance exercise. Men who are meeting current PA guidelines may be prescribed a vigorous intensity program, men who engage in aerobic or resistance exercise, but not enough to meet guidelines may be prescribed a moderate intensity program, while men who are inactive or sedentary will be prescribed a low intensity program.

This level contains the content in Level 1 plus personalized exercise and diet advice. Men in this group answer a series of questions about their exercise and diet habits and preferences at baseline to generate a personalized exercise and diet program. In addition, men in this group receive access to instructional exercise videos. The exercise programs generated for men are based on the evidence for exercise as a strategy to manage side effects and symptoms for prostate cancer, slow prostate cancer progression, reduce overall mortality risk, and/or improve physical functioning [9,10,19-21,27,57-62]. Tailoring of each program is based on a man’s response to a series of questions that ask about his health status, current and past prostate cancer treatments, health goals for exercise, current participation in aerobic and strengthening types of physical activity based on responses to the Rapid Assessment of Physical Activity questionnaire [63], resources for exercise (ie, access to an exercise facility, home exercise equipment, etc), and time available for exercise. A man’s stated health goals dictate the mode (aerobic and/or resistance training) of exercise training in his prescription; his exercise resources further dictate the type of training selected for his program (ie, machine weights in a gym vs resistance bands at home); his health status, prostate cancer treatments, and current exercise levels dictate the exercise intensity of his prescribed program; and his available time dictates the frequency and length of prescribed training sessions (Figure 1).

**Figure 2:**
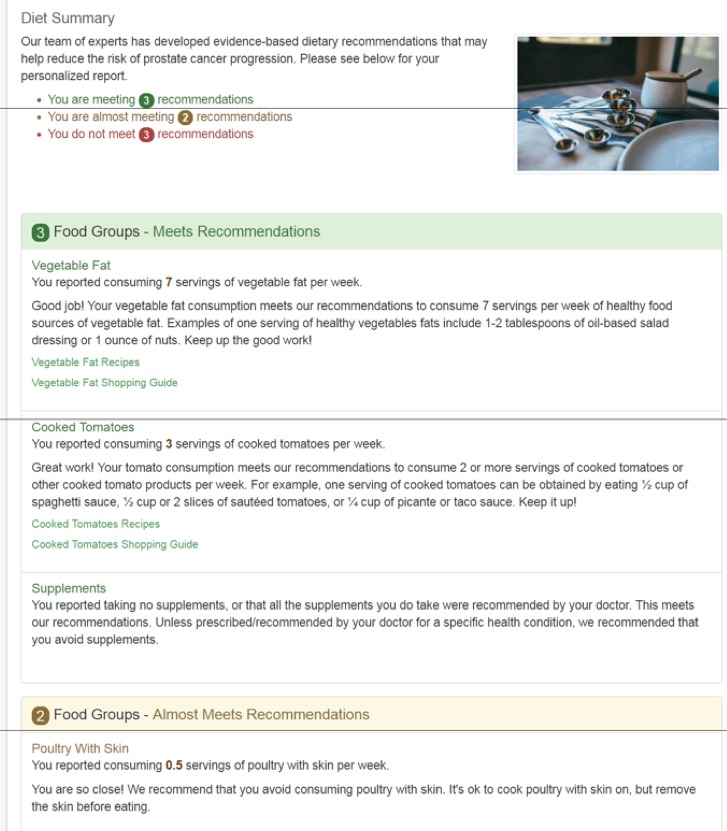
Example of the assessment and feedback that will be provided to a participant about how well he meets the dietary recommendations in the Community of Wellness.

Similarly, we assess men’s usual dietary habits using a validated food frequency questionnaire (FFQ) [64], benchmark his usual diet against 8 dietary recommendations we have developed based on the literature, and provide tailored advice on how he can improve his diet. Our recommendations are based on a review of the literature on diet and prostate cancer progression as of August 2016 [16] and also take into consideration recommendations from the American Cancer Society and dietary guidance provided for prevention of major chronic illnesses (eg, American Heart Association and American Diabetes Association recommendations). See Figure 2 for an example of how information would be provided back to participants about whether or not they are meeting dietary recommendations.

After receiving their personalized advice and access to instructional videos, men do not receive any further feedback to support behavior change.

#### Level 3: Teaching + Tailoring + Technology

This level contains the content provided in Levels 1 and 2 plus technology support for changing physical activity and diet habits. Men in this group are asked to log their diet and exercise behavior through the website and can view their progress over time. These men also receive a physical activity tracking device (Fitbit Alta; mailed to the participant) that interfaces with the Web portal and educational and motivational text messages about healthy diet and physical activity habits. Men are encouraged to use the Fitbit as a motivational tool and they can be synched to the portal where men can see displays of their time spent in physical activity, distance covered, and steps. Men do not receive separate physical activity goals related to use of their Fitbit. A total of 90 automated text messages are sent over the entire intervention period, averaging 4 texts per week. About 25% (23/90) of text messages ask the participant to reply to the message to promote an interactive experience.

#### Level 4: Teaching + Tailoring + Technology + Touch

**Figure 3:**
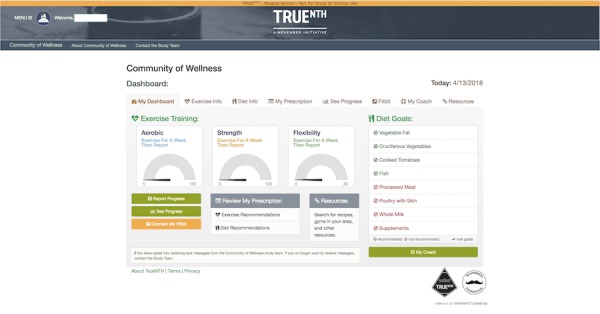
Example of a Level 4 “Dashboard” to help men navigate to different elements of the portal.

This level contains the content provided in Levels 1 to 3 plus interactive support via phone and online with research staff to support diet and exercise behavior change. Men in this group have the option to receive 2 30-min phone consultations, 1 with an exercise coach (certified athletic trainer) and 1 with a diet coach (registered cancer dietician). Participants also have the ability to receive ongoing advice from coaches via email sent through the Web portal. Men can email either coach as little or as often as they would like for the 12-week intervention period. Figure 3 provides a screenshot of a Level 4 dashboard.

### Randomization Assignment

The randomization scheme was for 4 arms, stratified by site (up to 5 sites were accommodated), with a block size of 4. The randomization sequence generation and randomization assignments were handled by study staff who were blinded to participants’ identifiers or any screening information. The randomization allocation sequence was generated by a staff statistician at the UCSF and shared with coinvestigators and team members at UCSF. Screening and enrollments were handled centrally by study team members at Oregon Health & Sciences University (OHSU). As patients enrolled, OHSU staff would request randomization allocations by site from UCSF staff by email; allocations were obtained by UCSF staff (who were blinded to patient identifying information) from the sequence following a consecutive order and communicated back by email to OHSU, typically within 24 hours. OHSU study staff then provided the appropriate Web intervention to each participant, based on the assignment received from UCSF. The randomization was done using *proc plan* in SAS v9.4.

### Safety

An important consideration for any program that delivers an exercise prescription or recommendation via the internet is ensuring participant safety. Information about how to perform the prescribed exercises and general tips for safe exercising are available on the Web portal. In addition, we have attempted to minimize the risk of adverse events during exercise by excluding men who may be at risk because of their current health status and/or prostate cancer treatment, unless they receive clearance from their physician to participate in moderate intensity exercise. In addition, the generated exercise prescription takes into account a man’s current health status, including whether or not he is currently receiving treatment for his prostate cancer with surgery, radiation or chemotherapy, ADT, or immunotherapy. For example, any man currently in active treatment is prescribed a low-intensity exercise program, regardless of other health indicators. If a man has no health indicators that dictate that he receives a low-intensity exercise program, the intensity of exercise is prescribed based on his self-reported baseline exercise levels so that the program is safe and effectively provides a sufficient stimulus for adaptation.

### Outcome Measures

We will evaluate acceptability and feasibility of the Community of Wellness intervention after 3 months via surveys, usage analytics from the Web portal, and focus groups. We will also assess study retention percentage of participants completing surveys) at 3 and 6 months as a reflection of acceptability and feasibility. Secondarily, we will explore the effectiveness of the different levels of the intervention on self-reported changes in diet or physical activity habits, QoL, and self-efficacy after 3 months and 6 months. To describe characteristics of our sample, we also ask men to self-report sociodemographic information (ie, age, race/ethnicity, marital, and employment status) and their cancer and health histories using an in-house questionnaire designed for this study.

#### Acceptability and Feasibility

To assess acceptability and feasibility of the Web-based intervention levels, we will evaluate accrual, participant retention, and adherence. We will measure accrual as the percentage of the target sample enrolled within a 1-year recruitment period. We expect to enroll the full sample (n=200) within this time frame. We will also collect information to allow us to determine the feasibility of recruitment, for example, percentage of men eligible and percentage of men enrolled out of the total contacted about the study. We will measure retention as the percentage of participants who complete postintervention and postfollow-up surveys out of the total sample. We expect to retain 80% of the full sample postintervention (n=160) and 80% of the postintervention sample (n=128) at follow-up. We will measure adherence as the number of times a man accesses the Web portal during the intervention period (quantified as the number of log-ins). We expect 100% of men to adhere to a single log-in and that the number of log-ins will significantly differ across levels, with Level 1 having the lowest and Level 4 having the highest adherence. We will measure the technical usage statistics for the Web portal, including frequency of log-ins, individual page views, and access of exercise videos. Post intervention, participants in each level are also asked to assess their use and the helpfulness of the Web portal, its components, and other study tools through an online survey.

#### Adherence to Diet and Exercise Recommendations

To estimate the effect of the intervention on behavior change, we will compare item-level data (eg, minutes per week of moderate activity, minutes per week of vigorous activity, and servings per week of cruciferous vegetables) at baseline and 3 months from the Community Healthy Activities Model Program for Seniors (CHAMPS) physical activity survey [65,66] and a validated FFQ [67]. We will examine behaviors overall as part of a “behavior score” (see below) and with a focus on activities and dietary components that correspond to our recommendations.

The CHAMPS survey is administered at baseline, 3 months, and 6 months. This validated survey estimates total weekly energy expenditure (in kilocalories per week) from physical activities for older adults. The 41-item questionnaire asks about recent engagement in specific types of vigorous-, moderate-, and light-intensity activities per week and the frequency and duration of participation. Specific items can be categorized as aerobic or resistance exercise, and responses to these items will be evaluated to determine how closely a man complied with his prescribed training program. In addition, we can calculate weekly energy expenditure in moderate- and/or vigorous-intensity activities separately for comparison with other studies.

The validated Harvard TH Chan School of Public Health FFQ for adults from 2007 is administered at baseline, 3 months, and 6 months [68]. This 132-item survey assesses dietary intake in categories from “never or less than once per month” to “6 or more times per day.” The original survey asks about intake over the past year; we modified the survey for this study to ask about intake over the last month.

In addition to the responses to standard surveys, participants who receive technology support (Levels 3 and 4) complete weekly online surveys to report their progress toward meeting their exercise and diet recommendations. Participants report the number and type (eg, aerobic or resistance) of exercise sessions, average session duration, and exercise intensity. Men also report which of the recommended dietary habits they worked on, if they met any of diet goals, and which goals were the most challenging. This type of charting is used to provide another form of behavioral support; we can also use the data to further evaluate adherence to recommendations.

We will calculate a “behavior score” based on how well each participants’ self-reported diet and exercise habits match the 8 dietary and 3 exercise recommendations. For each of the 11 targeted behaviors, participants will be assigned 0 points if they do not meet that recommendation, 1 point if they almost meet the recommendation, and 2 points if they meet the recommendation. The overall score ranges from 0 to 22, with higher values indicating closer adherence to the exercise and diet recommendations. To calculate the score, a priori numeric criteria were defined for not meeting, almost meeting, or meeting each of the 11 recommendations. These criteria were used in the design of the intervention to provide tailored feedback to participants randomly assigned to Levels 2 or above. For example, the recommendation for fish was to consume 2 or more servings per week. Men consuming less than 1 serving per week are considered “not meeting” the recommendation and receive 0 points toward the composite score; men eating more than 1 serving per week but less than 2 servings per week are considered “almost meeting” and given 1 point; and those eating more than 2 servings per week are “meeting the recommendation” and given 2 points. We will examine mean scores for each group at baseline and 3 months and changes in scores from baseline to 3 months. The development and application of a behavior score like this comes from literature examining healthy lifestyles and risk of chronic disease. For example, members of our team previously reported the development and validation of a lifestyle score for the prevention of lethal prostate cancer, which incorporated several of the diet and exercise components recommended in this study [69].

Other “healthy lifestyle” scores, computed similarly, have been developed and shown to be useful for summarizing lifestyle-disease relationships in other disease areas, such as hypertension or cardiac death [70,71].

To assess adherence to use of technology support via physical activity tracking devices for men in Levels 3 or 4, we will assess the number of days they wore their wearable device during the study period. We will also consider data from the wearable device to describe the average number of steps taken per day, active minutes per week, and miles covered per week.

#### Fatigue and Sleep Quality

Fatigue and sleep quality are assessed using validated instruments at baseline, 3 months, and 6 months. The Patient Reported Outcome Measurement Information System 7-item Short Form-Fatigue questionnaire [72] is used to assess changes in cancer-related fatigue. The Pittsburgh Sleep Quality Index is used to assess changes in sleep quality [73,74].

#### Self-Efficacy for Physical Activity and Diet

Self-efficacy for physical activity will be measured with a standard 6-item questionnaire at baseline, 3 months, and 6 months [75]. Self-efficacy for diet will also be collected at 3 and 6 months using a similar approach by having participants rate their confidence in performing each recommended task (eg, consume 2 or more servings of cooked tomatoes per week) using a Likert scale. We will evaluate whether baseline self-efficacy predicts response to the intervention arms and whether the interventions improve self-efficacy for physical activity and diet compared with control.

#### Stage of Change for Physical Activity

The Physical Activity Stage Assessment is a 5-item measure of a person’s readiness to engage in recommended levels of physical activity and has been used in prior studies of cancer survivors [47]. We will evaluate the influence of the behavioral support on readiness to engage in physical activity.

#### Focus Groups

Focus groups will be conducted post intervention to evaluate elements of usability and acceptability that will provide greater depth of information beyond what can be obtained via surveys. Focus group participants will be recruited from each of the different levels of intervention, to gain insight into participant experience at each of the levels. Focus groups will be conducted by phone so that we do not exclude participants who cannot travel to each study site. There is not an a priori selection of the focus group subsample; rather, we will examine the demographic data once each site is fully enrolled and contact a representative sample from the study population based on age, race/ethnicity, disease stage, time since diagnosis, and intervention arm.

### Statistical Considerations

#### Statistical Power

The primary outcomes for this pilot are acceptability and feasibility, as assessed by percent accrued in 1 year, percent recruited out of total contacted, and retention at 3 months. We anticipate enrolling 100% of the target 200 in 1 year, enrolling 3% of the total sample contacted via mass mailings, and retaining 80% of participants at 3 months. With 200 participants, we will be able to identify a retention proportion of 80% at 3 months (95% CI limits 74.5%-85.5%) and should be able to identify the other more extreme proportions with higher precision and tighter confidence limits. We will also assess the proportion completing surveys at 6 months, secondarily. If we assume 160 individuals complete the 3-month surveys, then we will have 80% power to detect a retention proportion at 6 months of 80% with a 95% confidence limit of 73.8% to 86.2%.

For the secondary outcome of behavior change, we will compute a composite *behavior score* at baseline and 3 months based on the self-reported survey data and as described above. With 50 participants per group, there is 80% of power to detect an effect size of 0.57 at a 2-sided alpha of .05 when comparing the change in score from baseline to 3 months for an intervention group (either Levels 2, 3, or 4) compared with the reference group (Level 1). For example, assuming on average men in the reference group (Level 1) experience a 2-point increase in scores from baseline to 3 months, 50 participants in Level 1 and 50 participants in Level 2 would provide power of 80% or higher if there is an average increase of 4.85 points or greater in Level 2, with an SD of 5. As a pilot study, multiple testing adjustments were not accounted for.

#### Proposed Statistical Analyses

We will examine the distribution of baseline covariates selected a priori (eg, age, race, clinical stage, body mass index, time since diagnosis, treatments received, current stage, and education level), overall and by group assignment. We will compute the time to enroll and proportions retained in the study (defined as completing at least one follow-up survey), overall and by arm, at 3 and 6 months. We will compare the dropout rates overall and across arms using chi-square or Fisher exact tests, as appropriate. Secondarily, we will examine the exercise and diet behavior data at baseline and 3 months for each study arm using descriptive statistics, such as means, medians, and interquartile range, and compare across arms using *t* tests and Wilcoxon rank sum tests. We will undertake an intent-to-treat analysis. We will also examine responses to the 6-month survey to explore retention and possible maintenance of behavior changes in longer term.

Our primary outcomes of this pilot RCT are accrual and participant retention. We will summarize eligibility, accrual, and retention using descriptive statistics and explore whether these factors differ by sociodemographic (ie, age, race/ethnicity, marital status, and employment) variables. In addition, we will examine adherence using the metrics described above (outcomes), overall and by randomization group.

We will explore changes in the secondary outcome metrics (eg, physical activity, diet, fatigue, and sleep quality) by describing data from baseline, 3 months, and 6 months for each study arm using descriptive statistics such as means, medians, and interquartile range. We will also examine whether change in physical activity and diet behavior, self-efficacy, fatigue, or sleep quality differs between the 4 levels of the intervention at baseline versus 3 months using *t* test, chi-square, analysis of variance, linear, and logistic regression methods, as appropriate. We will examine each group versus the reference (Level 1) as well as explore the hypothesis regarding whether increasing level of intervention corresponded to increase in uptake of the recommended behavior changes. We will consider primarily continuous variables when describing behavior change in each arm. For example, our first focus will be on whether individuals met, almost met, or did not meet the recommendations, and we will examine differences in the *behavior score* as described above. Next, we will consider the actual intakes of specific food groups of interest (eg, total servings of fish per week) and total minutes spent in each category of exercise (aerobic, strength, and flexibility) as continuous variables. We will also consider categorical variables to capture more broad indicators of positive behavior change using *i* cut points such as “increased in at least one dietary component, yes/no” or “increased in at least one exercise component, yes/no.” We will consider adjustment for important baseline covariates that may be associated with our outcomes, selected a priori [76]. We will also explore if exercise and diet self-efficacy and physical activity stage of change at baseline influence adherence to the intervention or the effect of the intervention on the secondary behavior change outcomes.

## Results

Development of the Community of Wellness website is complete, has been beta-tested, and is currently being evaluated in the RCT. This RCT is open for enrollment (ClinicalTrials.gov NCT03406013) and anticipates completing enrollment by the end of 2018. Recruitment is open at 3 of the 4 academic medical centers (OHSU, UCSF, UC Denver, and Emory University [pending]) with the target population of 50 men enrolled at each site for a total of 200 subjects. OHSU achieved their recruitment goal and enrolled 50 men. UCSF is actively recruiting, with 27 men currently enrolled, and UC Denver is just open. We expect to fully accrue to the trial by January 2019, with final data collection complete by July 2019.

## Discussion

We have developed a tailored, self-directed, and scalable Web-based intervention accessible via computer, tablet, or phone to promote lifestyle behaviors associated with better QoL and reduced risk of cancer recurrence/progression for men with prostate cancer. Although there are many general informational websites for cancer patients [77], the study Web portal provides a variety of tools and resources to empower prostate cancer survivors to improve their physical, emotional, and mental health, including tailored exercise and diet recommendations, contemporary technology-based support tools, and health coaching. A recent meta-analysis identified 15 internet-based programs that delivered a lifestyle-based (ie, physical activity and/or diet) intervention in cancer survivors [53]. An overall positive effect was found for increasing physical activity and reducing body mass index, but findings were mixed or inconclusive for cancer treatment–related symptoms, QoL, self-efficacy, and dietary change. Only 3 programs included, but were not specific to, men with prostate cancer. Thus, it is not known whether a Web-based program that delivers both exercise and diet advice and provides multiple levels of behavior change support is feasible, acceptable, and shows preliminary efficacy to improve diet and physical activity behaviors in prostate cancer survivors.

Although the internet is an increasingly common approach to building scalable programs for behavioral interventions, it remains unclear how Web-based programs should be designed to achieve the intended goal of behavior change. This pilot RCT will address this gap in our knowledge. We will determine the acceptability and feasibility, and estimate the effect on behavior, of 4 additive levels of Web-based tools, from Web-based information (Level 1) + personalized exercise and diet recommendations and training plans (Level 2) + text messages, a physical activity tracker (Level 3) + 1 phone counseling session with an exercise trainer and 1 session with a dietician (Level 4). A trade-off to adding tools is the resources required to implement and sustain them in an ever-changing technology environment. A purely technology-based program may also lack the interpersonal contact, which might be an important component for behavior change in men with prostate cancer [53]. Thus, in comparison with a control level of static educational content on physical activity and diet, this study will systematically evaluate layers of additional support for changing physical activity and diet behavior. Depending on the outcomes from our pilot, future research may include further optimizing of the Web portal using the Multiphase Optimization Strategy framework to identify which components of the intervention are driving behavior change and SMART (Sequential, Multiple Assignment, Randomized Trial) trial designs to determine how to optimally tailor these to the varying needs of men with prostate cancer [78].

## Abbreviations

CHAMPS: Community Healthy Activities Model Program for Seniors
FFQ: food frequency questionnaire
QoL: quality of life
RCT: randomized controlled trial
UCSF: University of California, San Francisco
